# Wheat and Flour

**Published:** 1876-10

**Authors:** 


*Wheat and Flour* exercise considerable influence upon the human organism so far as bone and muscle making materials are concerned, hence the following relating to these staples, which we find in the "Herald of Health," is interesting to dental practitioners.

Wheat is one of the most important articles of food, and probably used more extensively than any other grain. A kernel of wheat, as it comes out of the chaff or husk, is an interesting subject for thought. In a rough analysis it is composed of two parts, the colored, tough, tegumentary skin, and the easily pulverized interior, which we call flour. This tegumentary exterior layer is of so dense and woody a nature, and so tough, that it forms into a scale when ground. It is perfectly indigestible, and serves no purpose as a nutriment. Its effect on the bowels is to slightly irritate the alimentary canal, hurry the food through quickly, and with it valuable nutriment escapes without undergoing digestion. Beneath this external skin lies a soft, friable cortex, which in the ordinary process of grinding escapes with the tough, tegumentary portion. This part is very valuable nourishing material, as it is rich in nitrogenous matter, fat and salts. Bran, then, consists of two parts, the tough, worthless part, and that next to it, the very best part of the wheat. Still deeper in the kernel is another part, which is white, and composed mostly of starch, though there is also present some fat nitrogenous matter and salts. White flour, as ordinarily eaten, is composed of this inner white, and largely starchy substance.

Graham flour, on the other hand, contains all three parts, and is altogether to be preferred to the white, if made of good wheat, which contains a minimum of the tough cuticle. But within a few years a new method has been discovered of preparing flour from which this external cuticle has been removed, but not with it the layer just below it, which is really the most valuable part of the wheat, and which constitutes, as flour is generally ground, the only valuable part of the bran. Flour made in this way is vastly better than the flour with the bran removed ; for the latter is too starchy, and not sufficiently rich in nitrogenous, fatty and mineral matter. It is also much to be preferred to flour made with the whole of the external cuticle retained, for this is not nourishing, nor in any way useful, and may be is often injurious. We hail all improvements in flour as real benefactions to our race. We hailed the advent of whole wheat flour in preference to the superfine white, as it is more nourishing, and though objectionable on account of the tough, tegumentary portion, it is rich in gluten, fat, and mineral matter, We hail now again the new improvement, because it makes the perfect flour, free, as it is, from the excess of starch and deficiency of mineral matter, and free also from the woody skin of the wheat.

